# Final height prediction of girls at menarche: a combined model using left hand and wrist bone age, knee radiomic scores, and clinical characteristics

**DOI:** 10.1007/s12519-025-01002-5

**Published:** 2025-12-13

**Authors:** Xue-Qiong Xu, Yao Chen, Yi-Rou Wang, Fei-Han Hu, Juan Li, Guo-Ying Chang, Xin Li, Rui Wang, Yu Ding, Xiu-Min Wang

**Affiliations:** 1 Department of Endocrinology, Genetics and Metabolism, Shanghai Children’s Medical Center, Shanghai Jiao Tong University School of Medicine, Shanghai, 200127 China; 2 Department of Cardiology, Shanghai Children’s Medical Center, Shanghai Jiao Tong University School of Medicine, Shanghai, 200127 China

**Keywords:** Bone age, Final height, Knee, Menarche, Radiomic scores

## Abstract

**Background:**

Accurate final height prediction for girls with menarche is important, yet traditional Greulich–Pyle (GP) and Bayley–Pinneau predictions based on left hand-wrist bone age (BA) and target height demonstrate limited accuracy. This study aims to develop a method to more accurately predict final height.

**Methods:**

One hundred and seventy-three girls with menarche from August 2018 to June 2023 were analyzed retrospectively. BAs in Greulich and Pyle and Hoerr knee atlases were evaluated. Knee radiomic features were extracted using PyRadiomics; least absolute shrinkage and selection operator regression was utilized to develop radiomic scores of the distal femur and proximal tibia. Ordinary least squares regression with stepwise selection was used to build a multilinear equation. This was further compared with traditional methods in fivefold cross-validation (CV = 5) using residual distribution and Bland–Altman agreement analysis.

**Results:**

Height gain in our Chinese cohort after menarche was 8.94 ± 2.99 cm. A stepwise multilinear equation was built with height at menarche, BA of GP and radiomic score of the distal femur (*R*^2^ = 0.733, *F* statistic = 115.1,* P* < 0.05). Compared with traditional methods, a multilinear equation displayed the lowest residuals (residual range: − 5.677 cm to + 6.444 cm) and best Bland–Altman agreement (the mean difference: − 0.01 cm, 95% limits of agreement: − 3.96 to + 3.93 cm).

**Conclusions:**

A robust linear regression model that incorporates knee radiomic scores, BA of GP, height at menarche, and father’s height demonstrated the best final height prediction in our cohort. This research is an innovative application of radiomic score of the distal femur to final height prediction. Further validation is warranted to test robustness across populations and scenarios.

**Graphic abstract:**

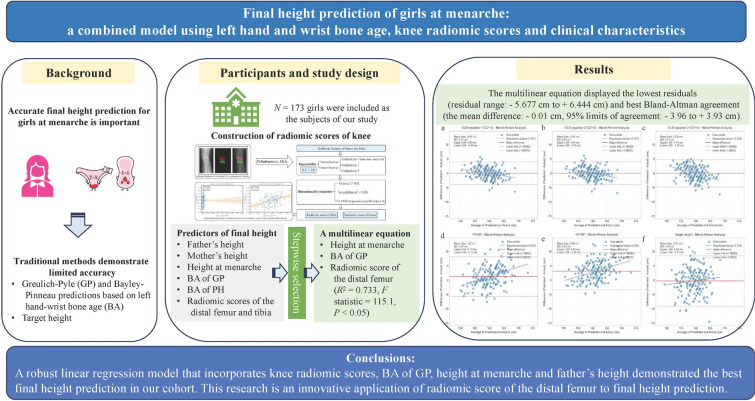

**Supplementary Information:**

The online version contains supplementary material available at 10.1007/s12519-025-01002-5.

## Introduction

The onset of menarche is a significant milestone in female pubertal development [1]. The duration of height growth after menarche is approximately 2 years, which signifies the final opportunity for height intervention [2–4]. Conventional wisdom indicates that girls experience growth of 5–8 cm following menarche [2, 3, 5, 6]. However, some studies have reported a wider range of variability in post-menarcheal growth, with standard deviations spanning 2.7 to 10.9 cm [2, 3, 5].

Accurate final height prediction for girls with menarche is essential for decision on height intervention. Current clinical methods primarily rely on left hand and wrist bone age (BA) assessed using the Greulich–Pyle (GP) atlas, often using the Bayley–Pinneau (BP) method for final height prediction [7, 8]. The GP-BP method has demonstrated limited predictive accuracy for final height prediction, with only 76.0% of predicted values within 5 cm of the actual measurements [9].

However, height is primarily determined by the length of the lower limbs. Individuals with greater final height tend to have proportionately larger leg length [10]. Average human height has also increased; this is largely associated with growth of the lower limbs [11]. The epiphyseal morphological changes in knee joint reflect growth of long bones in the lower limbs, which include the distal femur and proximal tibia. The distal femur accounts for 71% of femoral growth, while the proximal tibia contributes 57% [12]. Studies have shown that fusion of the knee joint epiphysis occurs later than left hand-wrist fusion; this probably better indicates growth potential for girls with menarche [13, 14].

Studies based on knee magnetic resonance imaging (MRI) confirmed the efficacy of knee joint images for predicting final height in children [15]. Effectiveness, high cost, and time-consuming nature of MRI limit widespread application in clinical settings. Evidence suggested that digital radiography (DR) images of the knee showed strong agreement with knee MRI in the evaluation of skeletal maturity [16]. Knee DR images may represent a practical and economical tool for height prediction. Like the GP atlas, the Pyle and Hoerr (PH) knee atlas originated from a longitudinal cohort study of the Brush Foundation. A classification system was subsequently designed to parallel the widely recognized GP atlas assessment framework [17].

Manual assessments of BA exhibited significant inter-observer variability, with up to 43% of predictions differing by more than 5 cm between observers using the traditional GP-BP method [18]. Recent advancements in artificial intelligence have demonstrated considerable promise in addressing the limitations of manual BA assessments, offering the capability to estimate skeletal maturity with accuracy and comparability to that of expert radiologists [19]. Radiomics represents an emerging quantitative approach in medical imaging which employs advanced computational techniques to extract extensive features from medical images [20]. Quantifying characteristics within radiomic images offer the potential for more objective assessments with reduced observer bias.

The aim of the present study in Chinese girls with menarche was to develop knee radiomic scores based on left knee DR images and to develop a higher accuracy final height prediction method when compared to the traditional GP-BP method.

## Methods

### Study population

**Fig. 1:**
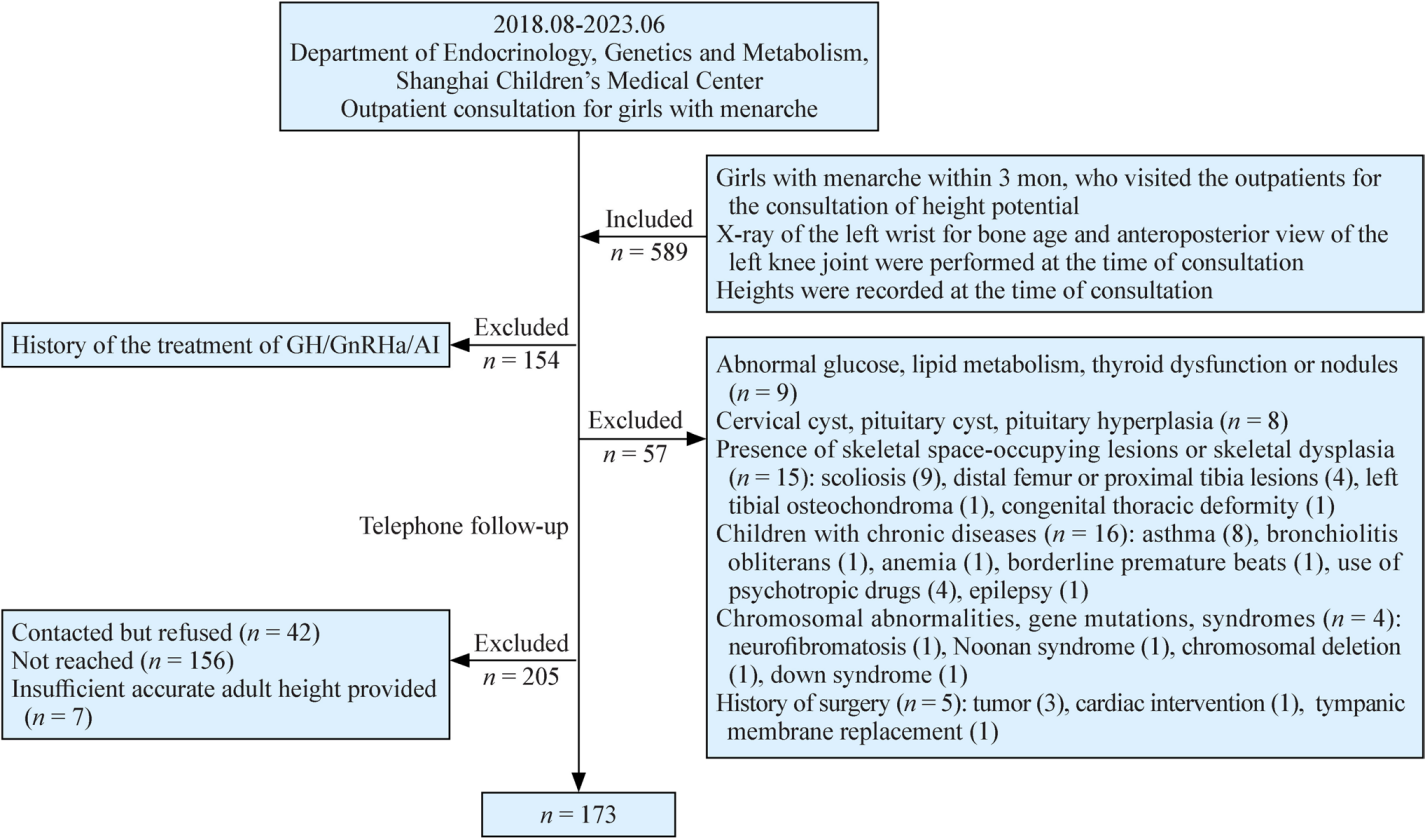
Study flow diagram. *GH* growth hormone, *GnRHa* gonadotropin-releasing hormone analogs, *AI* aromatase inhibitors

Subjects in this study were girls with menarche within the last 3 months, who visited our outpatient between August 2018 and June 2023. To be included in this study, participants needed to meet the following criteria: (1) menarche within the last 3 months and visited the outpatient Department for height potential consultations; (2) DR images of the left wrist along with anteroposterior views of the left knee were performed at the time of consultation; and (3) heights were recorded at the time of consultation. Subjects were excluded from this study based on any of the following criteria: (1) a history of treatment with any of the following, growth hormone (GH), gonadotropin-releasing hormone analogs (GnRHa) or aromatase inhibitors (AIs); (2) history of other endocrine or systemic diseases; or (3) an inability to obtain final height. Patient enrollment and distribution workflow is illustrated in Fig. 1.

Based on a comprehensive literature review and clinical evidence, we determined that height growth enters a plateau phase more than 2 years after menarche. Subsequent annual height gain decreases to less than 1 cm per year, which is considered clinically insignificant [21]. Therefore, we operationally defined final height as that measured 2 years after menarche.

Data from physical examinations were obtained from hospital medical records. Height (in centimeters) was measured using a calibrated Seca 274 stadiometer (Hamburg, Germany; precision: 0.1 cm) by trained pediatric assistants with more than 1 year of clinical experience. Girls and accompanying parents were measured in standardized conditions, that is wearing light clothing (t-shirts and shorts) without footwear. Measurements were made twice and the mean was calculated. Final height follow-up data were measured by parents with prior instruction on standardized height measurement to ensure data reliability. This was then reported to the study team.

### Prediction of children’s target height

Prediction of a child’s target height from the heights of their parents is essential in the estimation of growth. To predict a daughter’s height, target height is defined as the child’s mid-parental height, adjusted for sex by subtracting 6.5 cm [22].

#### Bone age assessment (left hand-wrist and left knee radiographs) and final height prediction using the Bayley–Pinneau method

BA assessment was performed using standardized radiographic atlas methodology. The GP atlas was used to evaluate BA from left hand-wrist radiographs, while the PH atlas was used for left knee radiographs. All BA evaluations were independently conducted by two experienced pediatric endocrinologists (CY and WYR), with the final BA determination representing the mean value of their assessments.

In addition, final height prediction was calculated using the conventional BP atlas [8]. This approach is based on the observation that, at any given chronological age, children will have attained on average a given percentage of their final height. Dividing a child’s present height by the average percentage of their mature height attained at that age yields an estimate of adult height.

#### Construction of radiomic scores of the distal femur and proximal tibia based on left knee radiographs

**Fig. 2:**
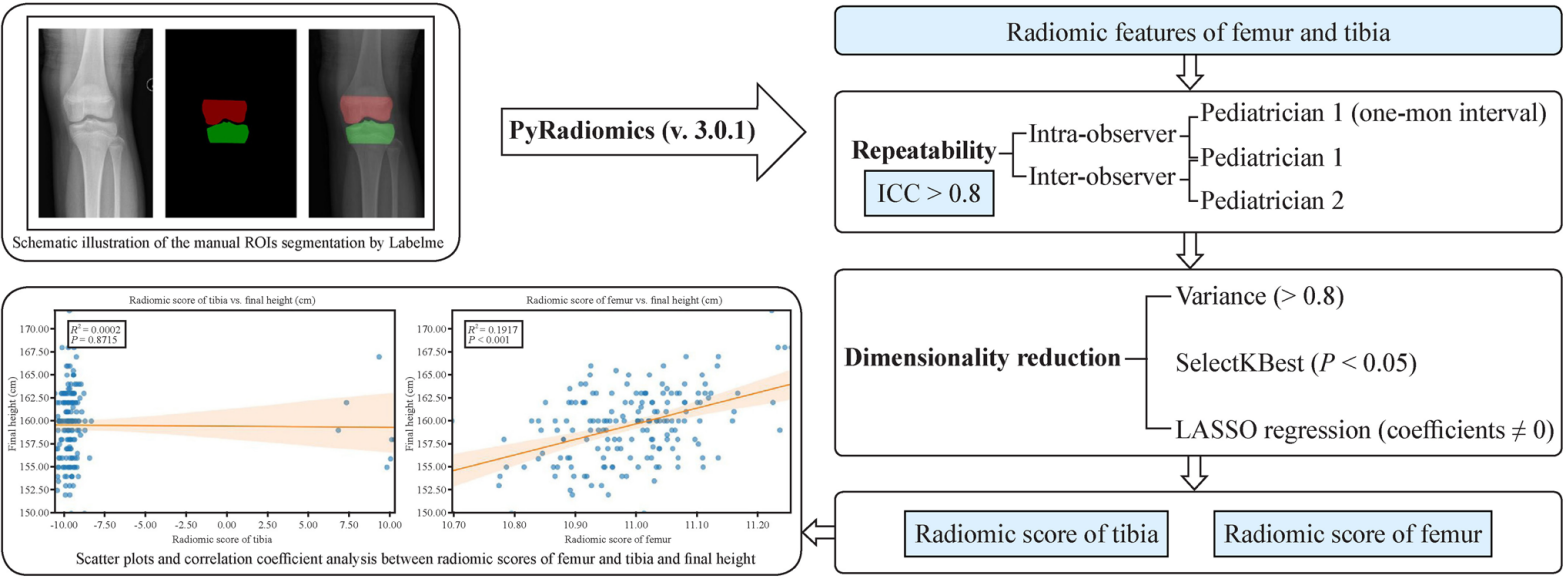
Radiomic score construction for femur and tibia. *ROIs* regions of interest, *ICC* intraclass correlation coefficient, *LASSO* least absolute shrinkage and selection operator

X-ray images of the left knee, originally in DICOM format, were exported as JPG images for analysis. Regions of interest (ROIs) were delineated in the distal femoral epiphysis and the proximal tibial epiphysis (Fig. 2). Labelme (version 5.4.1) was used to delineate these ROIs, while PyRadiomics (version 3.0.1) was employed to extract radiomic features within Python (version 3.10.11) [23]. Radiomic features both capture and provide rich information about shape, brightness, and texture of ROIs in medical images [24]. Shape features describe area, volume, perimeter, contour irregularity, and compactness. Texture features can be categorized as follows: first-order, second-order, and higher order statistics, and transform-based features. First-order features are also called histogram features. They provide the simplest level of information and are based on the distribution of individual pixel/voxel values within the lesions without emphasis on their spatial relationships. Second-order features are also called gray-level co-occurrence matrix features, and contain more information about texture by considering relationships between intensity of pairs of neighboring pixels/voxels. Higher order features go one step further and provide sophisticated patterns and textural information by emphasizing relationships between multiple pixels/voxels. Gray-level run length matrix, neighboring gray-level dependence matrix, neighborhood gray-tone difference matrix, gray-level size zone matrix, and gray-level distance zone matrix are examples of higher order features. Transform-based features evaluate textures and gray-level distribution within lesions in a different space after image transformation or filtering. We also employed wavelet transform, a widely-used image transformation technique for feature extraction.

Delineation was performed manually by two pediatricians, XXQ and WR. Repeatability of radiomic features was assessed via 60-case inter-observer (XXQ and WR) and 100-case intra-observer (XXQ, 1-month interval) evaluations. Reliability was evaluated using the intraclass correlation coefficient (ICC). Only radiomic features that demonstrated excellent stability, with ICC values exceeding 0.8, were selected for subsequent dimensionality reduction analysis (Fig. 2). Next, the dimensionality reduction procedure for radiomic features was systematically implemented across the entire sample cohort in three stages. The first stage involved selecting features with a variance over 0.8. SelectKBest, a filter-based feature selection method in scikit-learn, evaluates all features using a specified scoring function and selects based on the smallest *P* values and highest scores. In the second stage, features with a *P* value below 0.05 were selected using the SelectKBest method (Supplementary Fig. 1a and 1c). Finally, LASSO regression was employed to identify variables with non-zero coefficients (Supplementary Fig. 1b and 1d), facilitating the construction of radiomic scores for the femur and tibia (Table 2 and Fig. 2). LASSO regression is a method based on least absolute shrinkage and selection operator and is used in regression analysis for variable selection and regularization.

#### Ordinary least squares regression with stepwise selection: construction of equations and final height prediction

Our objective was to assess the potential use of radiomic scores of the distal femur and proximal tibia to aid prediction of final height for girls with menarche. As predictors of final height, we used father’s height, mother’s height, height at menarche, BA of GP, BA of PH and radiomic scores of the distal femur and tibia. First, we calculated correlations between predictors and final height (Supplementary Fig. 2). Then we used a stepwise algorithm to build a multilinear equation.

The algorithm started as a constant model. In each iteration, one independent variable was added to create an updated equation, which was tested for improved fit to the data. In each step, we chose the variable that was most strongly associated with the outcome measure and had a *P* value of at least 0.05. If the extended equation improved the fit (*P* value of the *F* statistic of the model fit below 0.05) and the added variable in the equation had a *P* value below 0.10, we accepted the updated equation. Otherwise, we excluded the variable and rejected the updated equation. For each equation, we calculated the total variance explained by the model, *R*^2^ and adjusted *R*^2^; *F* value and *P* value from the *F* test of data fitting of the updated equation versus the reduced equation. In addition, we evaluated model performance using the Akaike information criterion (AIC) and Bayesian information criterion (BIC), which balance model fit with complexity to guard against overfitting. Lower values of AIC and BIC indicate a more parsimonious and better-fitting model. To further assess the contribution of each predictor, we computed partial correlation coefficients (Table 3). To characterize the predictive value of ordinary least squares (OLS) models, we performed fivefold cross-validation (CV = 5), randomly selecting one-fifth of the data as the validation set. With the remaining four fifths of the data, we constructed a model using the variables identified in our stepwise algorithm. We computed predicted final heights for all children in the validation set.

### Statistical analysis

We calculated the goodness of fit (*R*^2^), adjusted *R*^2^ (corrected for predictor degrees of freedom), *F* value, and *P* value from the *F* test of overall significance in regression analysis (Table 3). To facilitate equation comparison, hierarchical hypothesis testing was conducted. For nested models, *F* tests were conducted to evaluate the statistical significance of incremental variance explained by additional variables, whereas likelihood ratio tests (LRT) were performed to compare the increases in *R*^2^ among equations. The AIC asymptotically selects the model that minimizes mean squared error of prediction or estimation and minimizes maximum possible risk in finite sample sizes. BIC tends to select the true model as the sample size grows. Partial correlation coefficients, which quantify the linear relationship between an independent variable and outcome while controlling for the effects of other variables in the model, provide insights into the direct association of each predictor with the dependent variable, independent of confounding influences. To assess robustness, we compared OLS and Huber’s robust regression results, examining coefficient consistency and significance levels (Supplementary Table 1). Robust regression methodology reduces the influence of outliers.

**Fig. 3:**
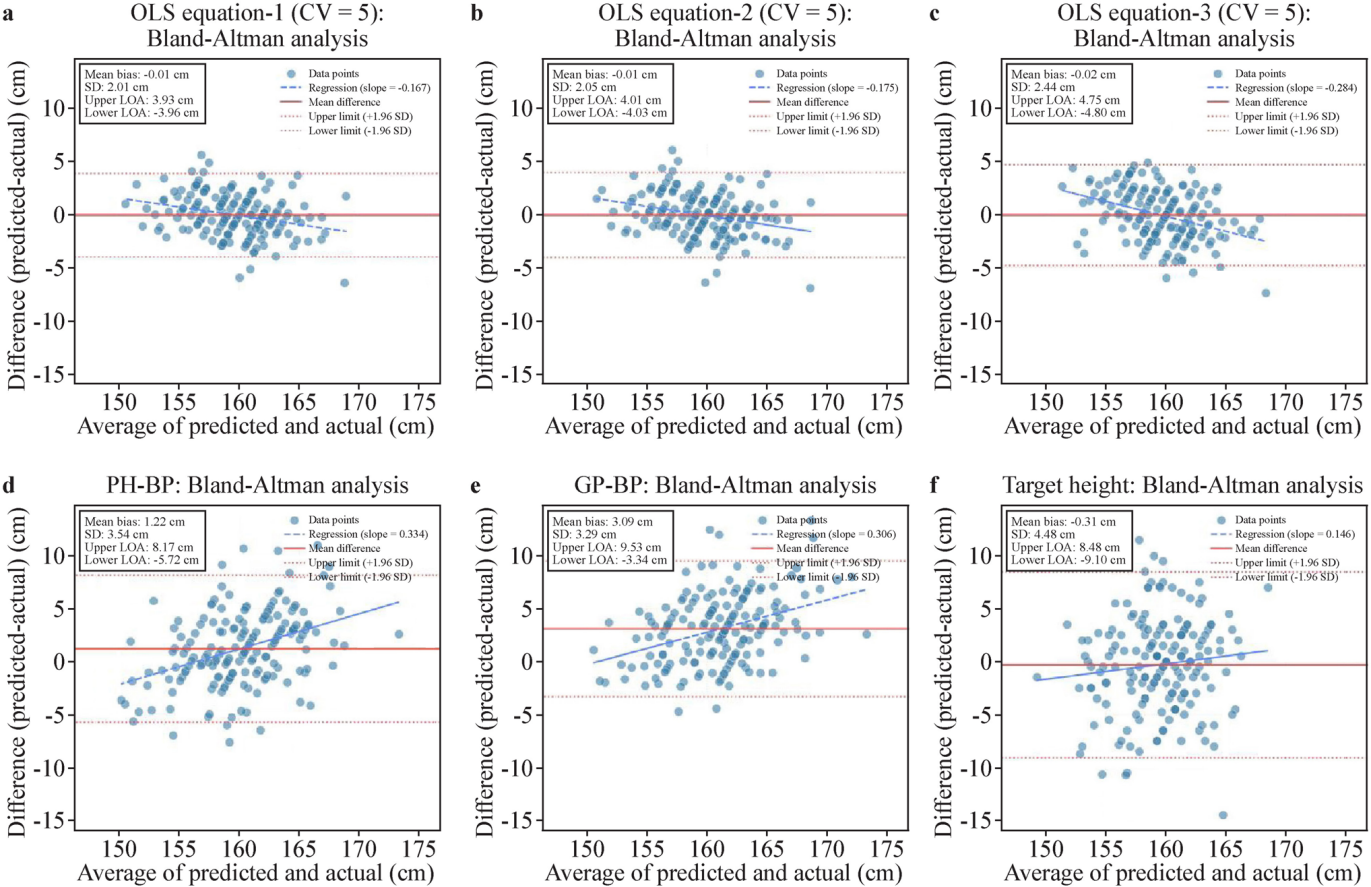
Bland–Altman agreement analysis for OLS equations (CV = 5), PH-BP, GP-BP, and target height. OLS equation-1: with independent variables of left hand-wrist bone age (BA) based on GP, radiomic score of femur, father’s height, and height at menarche. OLS equation-2: with independent variables of BA based on left hand-wrist GP, father’s height, height at menarche. OLS equation-3: with independent variables of radiomic score of femur, father’s height, and height at menarche. PH-BP, BA based on left knee PH atlas and BP method for final height prediction. GP-BP, BA based on left hand-wrist GP and BP method for final height prediction. Target height, defined as a child’s mid-parental height, by subtracting 6.5 cm to predict a daughter’s height. **a** Bland–Altman agreement analysis for OLS equation-1 (CV = 5); **b** Bland–Altman agreement analysis for OLS equation-2 (CV = 5); **c** Bland–Altman agreement analysis for OLS equation-3 (CV = 5); **d** Bland–Altman agreement analysis for PH-BP method; **e** Bland–Altman agreement analysis for GP-BP method; **f** Bland–Altman agreement analysis for target height method. CV = 5: fivefold cross-validation. *OLS* ordinary least squares, *LoA* limits of agreement, *GP* Greulich–Pyle [7], *PH* Pyle and Hoerr [17], *BP* Bayley–Pinneau [8], *SD* standard deviation

We evaluated model performance by calculating predicted height, *R*^2^ and root mean square error (RMSE) using fivefold cross-validation for the OLS models (Tables 4 and 5). To rigorously evaluate the performance of different height prediction methods, Bland–Altman agreement analysis was performed to quantify measurement concordance, determining both systematic bias (mean difference ± 1.96 standard deviation) and 95% limits of agreement (LoA) between predicted and final height (Fig. 3).

All analyses were performed within a Python (version 3.10.11) environment utilizing statistical and machine learning libraries. The threshold for statistical significance was set at *α* = 0.05 (two-tailed).

## Results

### Patient characteristics

From August 2018 to June 2023, a total of 589 girls visited the Department of Endocrinology, Genetics and Metabolism of Shanghai Children’s Medical Center for height potential consultations within 3 months of menarche. X-ray images of the left wrist and anteroposterior knee were taken. Heights of these girls were recorded at the time of the consultation. One hundred and fifty-four girls were excluded due to a history of GH, GnRHa or AI treatment. Fifty seven girls were excluded for other systemic diseases and chromosomal abnormalities, and two hundred five girls were excluded for the failure to complete telephone follow-up. Finally, 173 girls were included as the subjects of our study (Fig. 1).

Demographic characteristics of the study participants are presented in Table 1. Median age at menarche was 10.95 years, with an interquartile range (IQR) of 10.34 to 11.55 years. Median height gain was 8.70 cm (IQR: 6.9–10.50 cm). No statistically significant differences were identified between experienced pediatric endocrinologists in their assessment of BA of GP at menarche (CY: 12.36 ± 0.82 years, WYR: 12.35 ± 0.83 years, *t* = 0.13,* P* = 0.89). Just under two percent (1.73%) of cases exhibited discrepancies of more than 1 year in BA of GP assessments. Mean BA of GP at menarche was 12.35 ± 0.79 years. However, there were statistically significant differences between the experienced pediatric endocrinologists in their assessment of BA of PH at menarche (CY: 12.91 ± 0.65 years, WYR: 12.61 ± 0.66 years, *t* = 4.26,* P* < 0.01). Just over one percent (1.16%) of cases exhibited discrepancies of more than 1 year in BA of PH assessment. Mean BA of PH at menarche was 12.76 ± 0.58 years.

**Table 1:** Demographic characteristics of study participants (*N* = 173)

Characteristics	Minimum	Median (Q1, Q3)	Maximum	Mean ± SD
Age at menarche (y)	8.96	10.95 (10.34, 11.55)	14.23	11.01 ± 0.98
Height at menarche (cm)	138.00	150.40 (148.20, 153.50)	161.40	150.62 ± 4.28
Father’s height (cm)	155.00	172.00 (169.00, 176.00)	185.00	172.34 ± 5.46
Mother’s height (cm)	148.00	160.00 (156.00, 162.50)	175.00	159.10 ± 5.30
BA of GP at menarche (y)				
Pediatric endocrinologist 1	10.50	12.50 (12.00, 13.00)	15.50	12.36 ± 0.82
Pediatric endocrinologist 2	10.50	12.00 (12.00, 13.00)	15.00	12.35 ± 0.83
Mean BA of GP	10.50	12.25 (12.00, 12.88)	15.25	12.35 ± 0.79
BA of PH at menarche (y)				
Pediatric endocrinologist 1	11.00	13.00 (13.00, 13.00)	15.50	12.91 ± 0.65
Pediatric endocrinologist 2	11.00	13.00 (12.00, 13.00)	14.00	12.61 ± 0.66
Mean BA of PH	11.25	13.00 (12.50, 13.00)	14.75	12.76 ± 0.58
Final height (cm)	150.00	160.00 (157.00, 162.00)	172.00	159.56 ± 3.81
Height gain (cm)	2.10	8.70 (6.90, 10.50)	16.80	8.94 ± 2.99

Pediatric endocrinologist 1: author CY; pediatric endocrinologist 2: author WYR. *BA* bone age, *GP* Greulich–Pyle atlas based on left hand and wrist, *PH* Pyle and Hoerr atlas based on left knee [7, 17]. *Q1* first quartile, *Q3* third quartile, *SD* standard deviation

### Feature selection and radiomic score development of the femur and tibia

A total of 288 radiomic features were extracted from the distal femur and proximal tibia ROIs. Features with an ICC greater than 0.8 for both reviewers (XXQ and WR) were identified and resulted in 271 features for the distal femur and 266 features for the proximal tibia. For the distal femur, a variance threshold technique was utilized to extract 122 radiomic features; 71 of these features were selected through SelectKBest (Supplementary Fig. 1a). For the proximal tibia, a variance threshold technique was utilized to extract 122 radiomic features; 65 of these features were selected through SelectKBest (Supplementary Fig. 1c). Finally, LASSO regression using final height as the dependent variable identified non-zero coefficient features to construct radiomic scores for both regions (Supplementary Fig. 1b and 1d). Formulae for the distal femur and the proximal tibia radiomic scores are provided in Table 2. Integration of original imaging features with wavelet transform analysis, a multi-resolution spatial-frequency decomposition technique, has demonstrated critical value in constructing radiomic scores for femoral and tibial final height prediction. Of note, these texture features, which are imperceptible to pediatric endocrinologists, provide critical biomarkers for final height prediction.

**Table 2:** Formulae for radiomic scoring of the distal femur and proximal tibia

Anatomical site	Filters	Feature classes	Variables	Coefficient
The distal femur	–	Intercept	Intercept	160.0959
	Original	Firstorder	Mean	− 0.1090
	Wavelet-H	Glrlm	GrayLevelNonUniformity	1.0751
		Gldm	DependenceNonUniformity	0.4234
	Wavelet-L	Firstorder	Mean	− 0.1037
		Glrlm	RunLengthNonUniformity	0.0001
		Glszm	SizeZoneNonUniformity	0.0365
		Ngtdm	Complexity	0.5758
The proximal tibia	–	Intercept	Intercept	160.0959
	Original	Shape2D	MajorAxisLength	1.8547
			MaximumDiameter	− 2.4652
			Perimeter	1.1009
		Firstorder	10Percentile	− 1.0655
			Range	6.0021
			RobustMeanAbsoluteDeviation	1.4276
			Variance	− 0.0007
		Glcm	JointAverage	− 0.4492
			Glcm_SumAverage	− 1.072093e-13
		Glrlm_	LongRunEmphasis	0.2136
			RunLengthNonUniformity	3.8091
			ShortRunHighGrayLevelEmphasis	7.7804
		Glszm_	LargeAreaEmphasis	0.4598
			SmallAreaHighGrayLevelEmphasis	0.1444
			Complexity	− 4.1813
	Wavelet-H	Firstorder	Variance	− 4.5816
	Wavelet-L		InterquartileRange	0.6352
			Median	− 2.3973
			Minimum	6.3474
		Glcm	ClusterProminence	− 2.8356
		Glrlm	GrayLevelVariance	− 2.3664
			LongRunEmphasis	0.5052
			RunLengthNonUniformity	0.7373
		Glszm	GrayLevelVariance	− 0.0729
			LargeAreaEmphasis	0.2385
			SizeZoneNonUniformity	0.2360
		Ngtdm	Complexity	− 0.0861
		Gldm	DependenceNonUniformity	− 1.0041
			SmallDependenceHighGrayLevelEmphasis	− 0.8489

Formula = Sign(*S*) × log(∣*S*∣). *S* represents the linear combination of the intercept and the sum of the products of coefficients and variables. Sign(*S*) ensures that the sign of the radiomic score matches the sign of *S*. Log(∣*S*∣) is the natural logarithm of the absolute value of *S*. *glrlm* gray-level run length matrix, *gldm* gray-level dependence matrix, *glszm* gray-level size zone matrix, *ngtdm* neighborhood gray-tone difference matrix, *glcm* gray-level co-occurrence matrix

### Correlation between radiomic scores of the distal femur and proximal tibia and final height

We analyzed the relationship between radiomic scores derived from femur and tibia and final height. Scatter plots revealed distinct patterns for radiomic scores of the distal femur and proximal tibia. Radiomic score of the distal femur showed a significant positive correlation with final height (*R*^2^ = 0.19, *P* < 0.01). There was no statistically significant association between radiomic scores of the proximal tibia (*R*^2^ = 0.002, *P* = 0.87) (Fig. 2).

### Construction of final height prediction OLS regression using stepwise feature selection

Using final height as the dependent variable, we employed a stepwise selection algorithm (with entry *α* = 0.05 and retention *α* = 0.10) to identify significant predictors from seven candidate variables. The analysis yielded four significant predictors for our primary model (OLS equation-1*): height at menarche, BA of GP, and radiomic score of the distal femur (Table 3). To evaluate alternative predictive combinations, we also developed other two OLS equations. OLS equation-2 incorporated BA of GP, height at menarche, and father’s height. OLS equation-3 included radiomic score of the femur, height at menarche, and father’s height (Table 3).

**Table 3:** Summary of the OLS equations used for final height estimation

Variables	OLS equation-1*	OLS equation-2	OLS equation-3
Intercept			
Estimates	17.61 (− 18.07, 53.30)	57.76 (44.75, 70.77)	− 24.06 (− 66.00, 17.88)
* t* (*P*)	0.97 (0.33)	8.77 (**0.00**)	− 1.13 (0.30)
Height at menarche			
Estimates	0.70 (0.61, 0.78)	0.74 (0.66, 0.82)	0.54 (0.44, 0.63)
* t* (*P*)	15.80 (**0.00**)	18.42 (**0.00**)	10.93 (**0.00**)
Partial correlation coefficient (*P*)	0.77 (**0.00**)	0.82 (**0.00**)	0.64 (**0.00**)
Father’s height			
Estimates	0.09 (0.04, 0.15)	0.09 (0.03, 0.15)	0.15 (0.08, 0.22)
* t* (*P*)	3.14 (**0.00**)	2.91 (**0.00**)	4.12 (**0.00**)
Partial correlation coefficient (*P*)	0.24 (**0.00**)	0.22 (**0.00**)	0.30 (**0.00**)
Bone age of GP at menarche			
Estimates	− 1.91 (− 2.32, − 1.49)	− 2.00 (− 2.42, − 1.59)	–
* t* (*P*)	− 9.04 (**0.00**)	− 9.56 (**0.00**)	–
Partial correlation coefficient (*P*)	− 0.57 (**0.00**)	− 0.59 (**0.00**)	–
Femur radiomic score			
Estimates	4.07 (0.69, 7.45)	–	7.09 (3.06, 11.11)
* t* (*P*)	2.38 (**0.02**)	–	3.48 (**0.00**)
Partial correlation coefficient (*P*)	0.18 (**0.02**)	–	0.26 (**0.00**)
Metrics			
* R*^2^	0.733	0.724	0.603
Adjust *R*^2^	0.726	0.719	0.596
* F* statistic (*P*)	115.1 (**0.00**)	147.5 (**0.00**)	85.44 (**0.00**)
AIC	734.7	738.4	801.2
BIC	750.4	751	813.8
OLS equation-1* vs. OLS equation-2		*F* statistic = 5.67 (***P* = 0.02**), LRT: D = 5.74 (***P* = 0.02**)	
OLS equation-1* vs. OLS equation-3		*F* statistic = 81.71 (***P* < 0.01**), LRT: D = 68.57 (***P* < 0.01**)	

OLS equation-1* indicates the final stepwise equation. Estimates include the coefficient, with 95% confidence intervals in parentheses. Bold text indicates *P* < 0.05. OLS equation-1*: with independent variables of left hand-wrist bone age, radiomic score of femur, father’s height, and height at menarche; OLS equation-2: with independent variables of left hand-wrist bone age, father’s height, height at menarche; OLS equation-3: with independent variables of radiomic score of femur, father’s height, and height at menarche. *OLS* ordinary least squares, *GP* Greulich–Pyle atlas based on left hand and wrist [7], *LRT* likelihood ratio test, *R*^*2*^ coefficient of determination, *AIC* Akaike information criterion, *BIC* Bayesian information criterion

OLS equation-1 explained 72.6% of the variance (adjusted *R*^2^ = 0.726). Height at menarche, use of BA of GP method, father’s height, and radiomic score of the distal femur showed a strong correlation with final height (all *P* < 0.05). Moreover, OLS equation-1 exhibited a superior model fit compared to OLS equation-2 and OLS equation-3, as shown by a higher adjusted *R*^2^, lower AIC value, and lower BIC value; these were significant in *F* tests and LRT (Table 3).

Robustness checks revealed that the estimated relationships between key variables in regression analyses were consistent with our primary results, indicating methodological robustness in OLS equations-2 and 3 (Supplementary Table 1). In OLS equation-1, height at menarche, father’s height, and BA of GP were consistent, while femur radiomic scores were significant with *P* = 0.02 but marginal (*P* = 0.08) in robustness testing.

#### Prediction of final height based on target height, traditional BP methodology based on BA (GP and PH) and three OLS models in a fivefold cross-validation (CV = 5)

To evaluate the predictive accuracy of three OLS models in comparison with traditional methods (PH-BP, GP-BP, and target height), we conducted a fivefold cross-validation (CV = 5) analysis. Comparative analysis of final height prediction methods revealed significant performance differences between the OLS (CV = 5) and traditional methods.

We compared predicted final height values with actual final height (Table 4). Students’ *t* testing of the means revealed that the predicted final height values derived from both the PH-BP and GP-BP methods showed significant differences with the actual final height (*P* < 0.05). In contrast, the three OLS models and the target height did not differ significantly from actual final height.

**Table 4:** Predicted final height from three OLS equations (CV = 5), BP-PH, GP-BP, and target height

Final/predicted height (cm)	Final height	OLS equation-1 (CV = 5)	OLS equation-2 (CV = 5)	OLS equation-3 (CV = 5)	PH-BP	GP-BP	Target height
Minimum	150.00	151.04	151.44	151.19	148.36	150.16	148.50
Median (IQR)	160.00 (5.00)	159.41 (3.83)	159.48 (3.54)	159.52 (3.38)	161.04 (7.03)	162.66 (6.82)	159.50 (6.00)
Maximum	172.00	169.78	169.18	167.62	174.58	176.05	172.00
Mean ± SD	159.56 ± 3.81	159.54 ± 3.26	159.55 ± 3.23	159.53 ± 2.95	160.78 ± 5.08*	162.62 ± 4.98*	159.25 ± 4.20

OLS equation-1: with independent variables of left hand-wrist bone age (BA) based on Greulich–Pyle (GP) [7], radiomic score of femur, father’s height, and height at menarche; OLS equation-2: with independent variables of BA based on left hand-wrist GP, father’s height, height at menarche; OLS equation-3: with independent variables of radiomic score of femur, father’s height, and height at menarche. PH-BP, BA based on left knee Pyle and Hoerr (PH) atlas [17] and Bayley–Pinneau (BP) method [8] for final height prediction. GP-BP, BA based on left hand-wrist GP and BP method for final height prediction. Target height, defined as a child’s mid-parental height, by subtracting 6.5 cm to predict a daughter’s height. CV = 5: fivefold cross-validation. *OLS* ordinary least squares, *IQR* interquartile range, *SD* standard deviation. ^*^The means of the predicted height significantly differ from final height at a *P* value of 0.05

As shown in Table 5 and Fig. 3, the OLS models demonstrated superior predictive accuracy across all evaluation metrics. Among them, OLS equation-1 (CV = 5) achieved the best performance with an RMSE of 2.01 cm, *R*^2^ of 0.720, the mean difference of − 0.01 ± 2.01 cm, and 95% LoA of − 3.96 to 3.93 cm between predicted and actual height (Fig. 3a). OLS equation-2 (CV = 5) performed similarly well with an RMSE of 2.05 cm, *R*^2^ of 0.709, mean difference of − 0.01 ± 2.05 cm, and 95% LoA of − 4.01 to 4.03 cm (Fig. 3b). OLS equation-3 (CV = 5) maintained good accuracy though with slightly higher errors (RMSE = 2.44 cm, *R*^2^ = 0.588, mean difference of − 0.02 ± 2.44 cm and 95% LoA: − 4.75 to 4.80 cm, Fig. 3c).

**Table 5:** Summary of three OLS equations (CV = 5), BP-PH, GP-BP, and target height for final height prediction

Variables	OLS equation-1 (CV = 5)	OLS equation-2 (CV = 5)	OLS equation-3 (CV = 5)	PH-BP	GP-BP	Target height
*R*^2^	0.720	0.709	0.588	0.028	− 0.411	− 0.398
Residual (cm)						
Minimum	− 5.677	− 6.127	− 4.936	− 11.030	− 13.350	− 11.500
Median (IQR)	1.309 (2.660)	1.396 (2.557)	1.867 (3.206)	2.530 (4.650)	3.300 (4.480)	3.000 (5.500)
Maximum	6.444	6.932	7.384	7.630	4.720	14.500
Absolute residual ≤ 2 cm, %	67.6	66.5	52.6	42.3	31.8	39.3
2 < Absolute residual < 5 cm, %	30.1	30.6	45.7	39.9	42.2	35.3
Absolute residual ≥ 5 cm, %	2.3	2.9	1.7	17.9	26.0	25.4
RMSE (cm)	2.013	2.051	2.438	3.748	4.514	4.494
RPD	1.857	1.827	1.525	1.014	0.842	0.846

OLS equation-1: with independent variables of left hand-wrist bone age (BA) based on Greulich-Pyle (GP) [7], radiomic score of femur, father’s height and height at menarche; OLS equation-2: with independent variables of BA based on left hand-wrist GP, father’s height, height at menarche; OLS equation-3: with independent variables of radiomic score of femur, father’s height, and height at menarche. PH-BP, BA based on left knee Pyle and Hoerr (PH) atlas [17] and Bayley–Pinneau (BP) [8] method for final height prediction. GP-BP, BA based on left hand-wrist GP and BP method for final height prediction. Target height, defined as a child’s mid-parental height, by subtracting 6.5 cm to predict a daughter’s height. CV = 5: fivefold cross-validation; residual = final height − predicted height. *OLS* ordinary least squares, *IQR* interquartile range, *RMSE* root mean square error, *RPD* ratio of performance to deviation

In contrast, traditional methods exhibited substantially higher prediction errors. The GP-BP method systematically overestimated the actual height with mean difference of 3.09 ± 3.29 cm (RMSE = 4.51 cm and 95% LoA: − 3.34 to 9.53 cm, Fig. 3e). The target height method showed the broadest 95% LoA of − 9.10 to 8.48 cm (RMSE = 4.49 cm and mean difference of − 0.31 ± 4.48 cm, Fig. 3f). PH-BP method demonstrated intermediate accuracy (RMSE = 3.75 cm, mean difference of 1.22 ± 3.54 cm and 95% LoA: − 5.72 to 8.17 cm, Fig. 3d) but still underperformed compared to OLS models.

## Discussion

In this study, height gain observed in our cohort of Chinese girls after menarche was 8.94 ± 2.99 cm. Target height demonstrated the widest residual range and shows the poorest agreement with final height. For traditional BP methods, BA of PH exhibited superior accuracy compared to BA of GP. The OLS equation methods demonstrated superior performance compared to traditional methods. We successfully developed knee radiomic scores for Chinese girls with menarche. The optimal OLS model was constructed using stepwise regression with height at menarche, father’s height, BA of GP, and radiomic score of the distal femur. Compared with OLS equation-2, OLS equation-1 appended radiomic score of the distal femur as independent variables exhibited significantly increased *R*^2^ and less variance. These findings indicate that radiomic score of the distal femur could be an objective and reliable predictor of final height for girls with menarche.

Median age and height at menarche in our cohort were 10.95 years and 150.40 cm, respectively. These were similar to values of 10.7 years and 149.1 cm reported from 785 Chinese girls at the Capital Institute of Pediatrics [25]. The BA of GP at menarche observed in our cohort was 12.32 ± 0.78 years; this was slightly lower than a study in girls from Taipei at 12.8 years [26]. The BA of PH at menarche observed in our cohort was 12.76 ± 0.58 years. The observed BA discrepancies between GP and PH may stem from inherent variations in skeletal maturation rates across different anatomical sites or systematic differences embedded within the respective atlas standards themselves.

It is widely recognized that menarche has been occurring earlier in girls. In a study analyzing eight sub-Saharan African countries, it was found that greater gains in height were associated with a more rapid decline in the age at menarche [27]. It is unknown whether these trends will lead to an increase in growth potential after menarche in girls. The post-menarcheal increase in height observed in our cohort was 8.94 ± 2.99 cm, which is higher than reported in the existing literature. In a Brazilian study, the post-menarcheal height gain was 7.5 cm [2]. Similarly, in Chile, Singleton et al. reported that girls grew an average of 6.6 ± 2.5 cm after 4 years of menarche [4]. In addition, a study verified that stature differences between ethnic groups already existed at menarche [3]. This study offers valuable insights into Chinese girls’ post-menarcheal height gain, which appears more substantial than previously documented. However, several methodological limitations warrant cautious interpretation such as the restricted sample size and potential sampling bias of a single-center study.

Menarche represents the final window for height-modifying interventions, yet post-menarcheal growth potential varies significantly. While Avendaño et al. reported an average height gain of 6.4 ± 2.7 cm with three subjects growing 15 cm and one 22 cm [28]. These findings highlight the critical need for a precise method to predict final height for girls with menarche. Although the GP-BP method has been widely adopted in clinical practice, this study found GP-BP method overestimated final height. This systematic error likely stems from the fundamental assumption of the method that phalangeal growth patterns can reliably predict long bone development; a previous study showed mean errors up to 3.4 cm and confidence intervals as wide as 8 cm [15]. The GP method may produce an overestimation of BA in Asian girls aged 10–13 years with high inter-observer variability ranging from 0.45 to 0.83 years [29, 30]. Clinical interpretation of GP-BP method-based predictions requires caution.

OLS equation-2 (CV = 5) with independent variables of left hand-wrist BA, father’s height, and height at menarche performed similarly well with an RMSE of 2.05 cm. This demonstrated superior performance to GP-BP. In our study, two pediatric endocrinologists with over 5 years of specialized experience in BA assessment ensured expert-level reliability. No statistically significant differences were identified between these investigators in the assessment of BA of GP at menarche. However, BA assessment involves subjectivity in junior pediatric endocrinologists [31]. AI-assisted methods such as BoneXpert provide more accurate and reliable BA estimations compared to traditional methods like GP and are already in use. However, it is important to highlight that BA assessments from BoneXpert displayed the lowest agreement with GP over the age range 9 to 14 years. This encompasses the majority of subjects in our study [32].

The concentrated knee epiphyses (distal femur, proximal tibia) enable BA estimation and height prediction [16]. The PH-BP method demonstrated the highest predictive accuracy with 82% of estimates within 5 cm and minimum RMSE of 3.75 cm in traditional final height prediction methods. However, the inherent subjectivity in BA interpretation remains an unavoidable challenge. There were statistically significant differences between the experienced pediatric endocrinologists in the assessment of BA of PH at menarche. Clearly there is a critical need for an objective assessment.

MRI-derived knee data is potentially valuable and novel. Indeed, knee MRI tract volume has proven effective in predicting pediatric height gain and total growth [15]. While MRI is expensive, time-consuming, and unsuitable for outpatient assessments, a DR image of the knee, with an ultra-low radiation dose (< 0.00012 mSv), offered a convenient outpatient assessment [33]. Radiomics extracts quantitative image features from a DR image of the knee to enhance clinical decision-making [20], through dual-observer ROI delineation and only selecting radiomic features that demonstrated excellent stability (ICC > 0.8) for subsequent dimensionality reduction analysis. Our radiomic scores demonstrated superior objectivity. After feature selection, we successfully established a radiomic score for the distal femur which correlated with final adult height. This was attributed to the volume and surface area of the growth plate [34]. Through stepwise regression analysis, the radiomic score of the distal femur was identified as a key predictive variable and included in OLS equation-1. Radiomic scores of the distal femur correlated with final height in OLS equation-1 and OLS equation-3, suggesting that these quantitative imaging biomarkers may capture subtle maturation patterns not detectable through traditional BA assessment. Implementation of this framework exhibits significant clinical accessibility, as both the ROI delineation software (Labelme version 5.4.1) and Python-based feature extraction libraries (PyRadiomics version 3.0.1) are publicly available. The sole manual procedure requiring clinician input is distal femoral ROI annotation. This process has been rigorously validated through dual-observer analysis and demonstrates robust feature reproducibility and mitigates operator-dependent variability risks. Our study pioneers its application in final height prediction for girls with menarche through objective, reproducible knee DR image radiomic scores.

OLS equation-3 (CV = 5), with independent variables of radiomic femur score, father’s height, and height at menarche, maintained good accuracy. Although OLS equation-3 showed slightly reduced accuracy compared to OLS equation-1 and 2, it still outperformed all traditional methods. Of note, OLS equation-3 eliminates the inter-observer variability inherent in BA assessment, as it does not require radiographic interpretation by pediatric endocrinologists. This objective approach ensures consistent, operator-independent predictions that may be particularly valuable in resource-limited settings or when expert readers are unavailable.

Final height is influenced by genetic, environmental, and nutritional factors. The target height method showed the broadest 95% LoA of − 9.10 to 8.48 cm. In stepwise regression analysis, this study highlights the stronger predictive role of parental height, consistent with existing genetic theories [35]. Although maternal height also contributes to children’s height, the predictive power of paternal height was more pronounced in this study. This may be related to the expression of several sex-specific genes or could reflect other potential influences of fathers in the family environment, such as nutrition and lifestyle habits [36].

Moreover, this study revealed that stepwise regression-derived OLS equation-1, incorporating BA of GP, knee radiomic score of the distal femur, and clinical characteristics, demonstrated best accuracy in final height prediction for Chinese girls with menarche. The combination of both BA of GP and radiomic score of the distal femur was more accurate than using either parameter independently. To our knowledge, this represents the first investigation to integrate these two anatomical sites in final height prediction.

Our study had some limitations. First, the number of adolescents studied was small so our results may have some bias. Second, final height data was obtained by parents following standardized protocols. Although clear instruction was provided to ensure proper measurement, potential errors could occur. Third, caution should be exercised when generalizing these findings to the broader population, as the study specifically focused on individuals attending as outpatients for consultations; these may not fully represent the general demographic. Comparison of OLS models (CV = 5) with traditional methods is problematic statistically. This may have caused bias that fivefold cross-validation cannot correct. Future studies should focus on expanding the sample size and incorporating external validation cohorts to enhance the robustness and generalizability of the findings. Although radiomic score of the distal femur improved the OLS model, the effect size was modest suggesting potential underexplored nonlinear relationships; these should be explored.

In conclusion, when compared with traditional methods, a robust linear regression model that incorporates radiomic score of the distal femur, BA of GP, height at menarche, and father’s height demonstrated the lowest residuals for final height prediction in our cohort. Although performance in regression analysis of radiomic scores of the distal femur, BA of GP, height at menarche, and father’s height is inferior to that of BA of GP and its associated indicators, their predictive accuracy remained significantly better than traditional methods. More importantly, this approach eliminates the need for subjective BA, demonstrating distinct advantages in objectivity, efficiency, and convenience.

## Supplementary Information

Below is the link to the electronic supplementary material.

Supplementary file 1 (PDF 1273 KB)

Supplementary file 2 (PDF 241 KB)

Supplementary file 3 (PDF 454 KB)

Supplementary file 4 (PDF 253 KB)

## Data Availability

The datasets generated and/or analyzed during the current study are not publicly available but are available from the corresponding author on reasonable request.
