# Supplementary material for: Final height prediction of girls at menarche: a combined model using left hand and wrist bone age, knee radiomic scores, and clinical characteristics
Source: World J Pediatr. 2025 Dec 13;22(1):129–41. doi: 10.1007/s12519-025-01002-5 (PMC12894113; doi:10.1007/s12519-025-01002-5)
Supplement: Supplementary file 1 — (PDF 1273 KB) [file 12519_2025_1002_MOESM1_ESM.pdf]

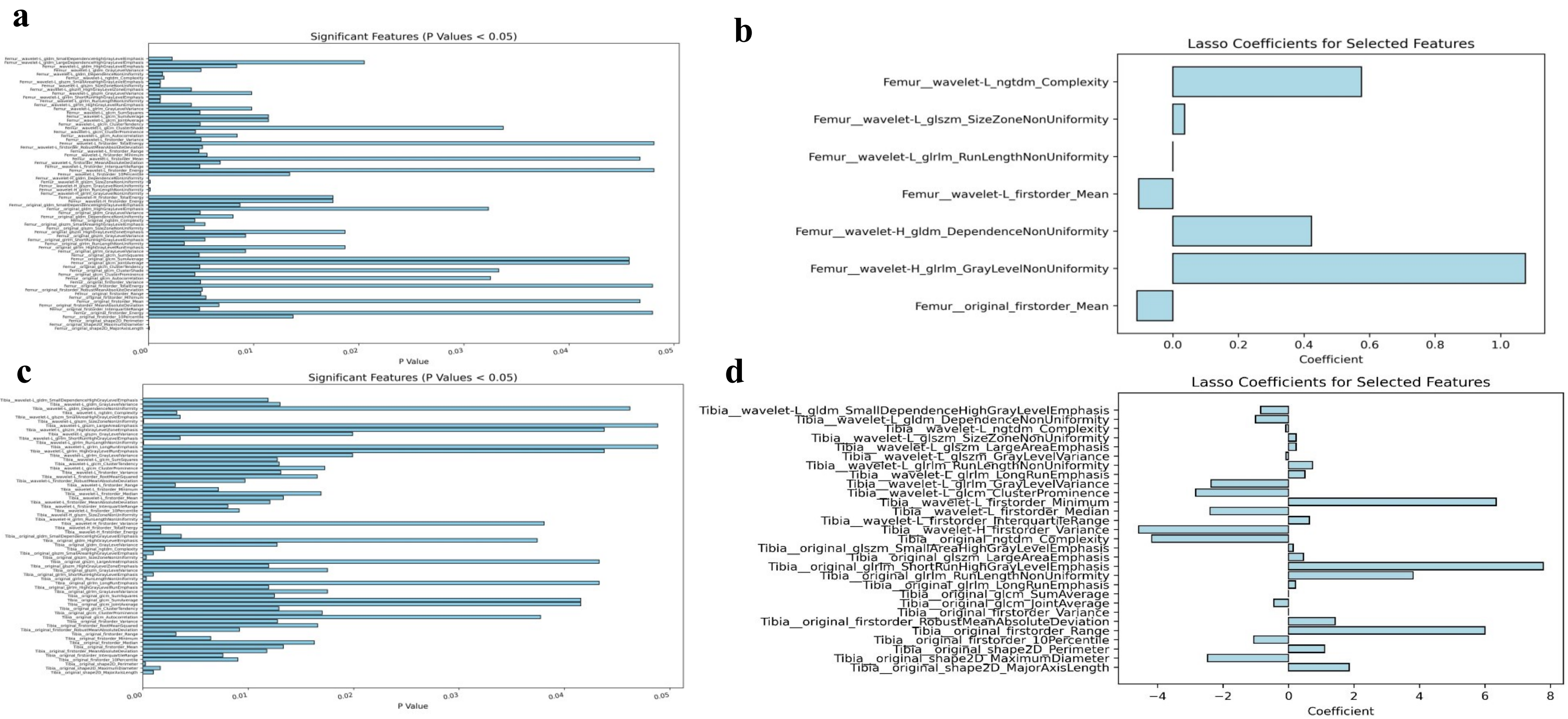

Supplementary Fig. 1 Dimensionality reduction of femoral and tibial radiomic features using SelectKBest and LASSO. **a** Statistically significant radiomic features (*P* < 0.05) selected by SelectKBest for the distal femur; **b** LASSO-derived feature subsets with non-zero coefficients for the distal femur radiomic score computation; **c** statistically significant radiomic features (*P* < 0.05) selected by SelectKBest for the proximal tibia; **d** LASSO-derived feature subsets with non-zero coefficients for the proximal tibia radiomic score computation. *LASSO* least absolute shrinkage and selection operator

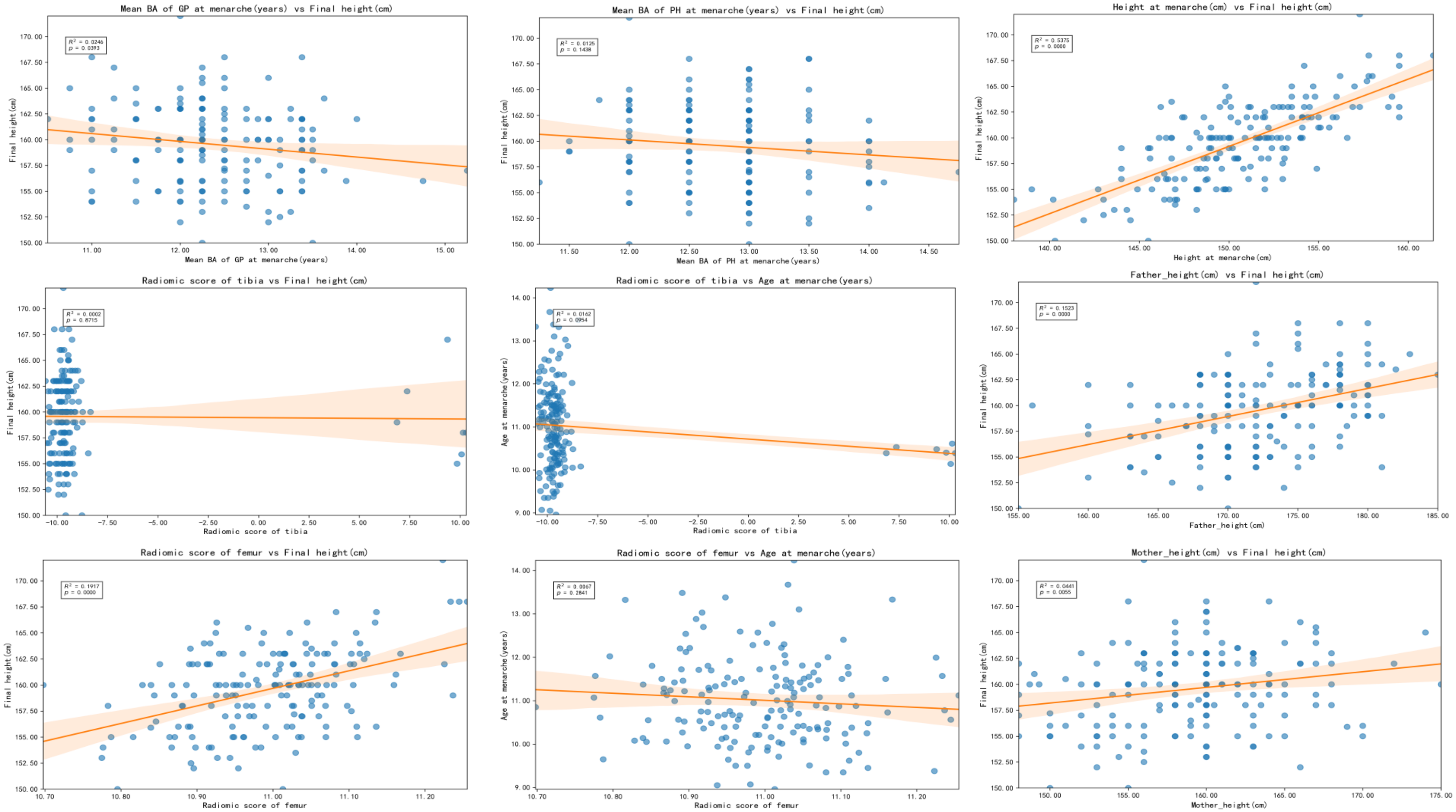

Supplementary Fig. 2 Correlations between radiomic scores of femur and tibia, bone age based on GP and PH, clinical characteristics and final height. *GP* bone age based on Greulich-Pyle (GP) left hand-wrist atlas, *PH* bone age based on Pyle and Hoerr (PH) left knee atlas [7,17]

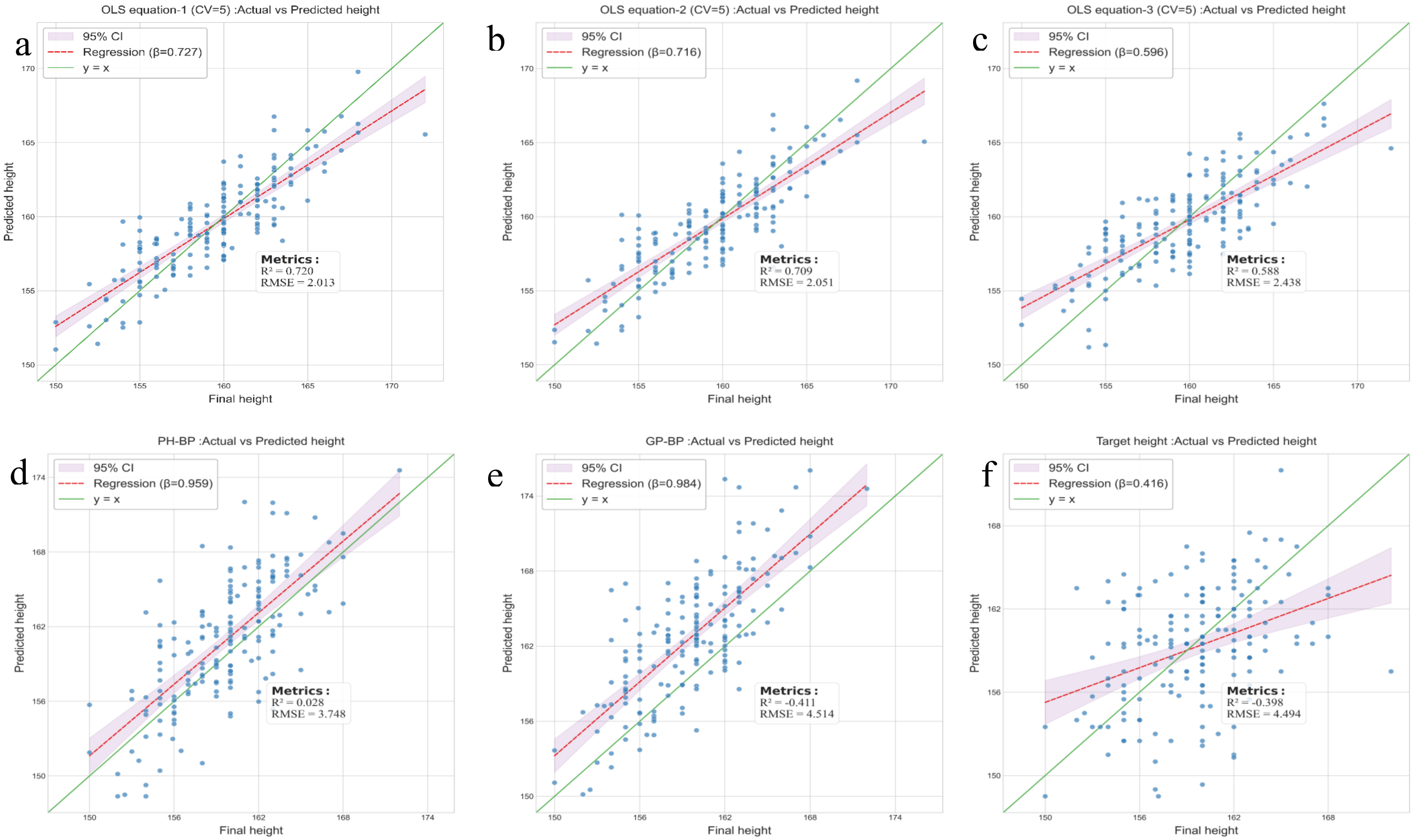
a
OLS equation-1 (CV=5) :Actual vs Predicted height
95% CI
Regression (β=0.727)
y = x
Metrics :
R² = 0.720
RMSE = 2.013
Predicted height
Final height
b
OLS equation-2 (CV=5) :Actual vs Predicted height
95% CI
Regression (β=0.716)
y = x
Metrics :
R² = 0.709
RMSE = 2.051
Predicted height
Final height
c
OLS equation-3 (CV=5) :Actual vs Predicted height
95% CI
Regression (β=0.596)
y = x
Metrics :
R² = 0.588
RMSE = 2.438
Predicted height
Final height
d
PH-BP :Actual vs Predicted height
95% CI
Regression (β=0.959)
y = x
Metrics :
R² = 0.028
RMSE = 3.748
Predicted height
Final height
e
GP-BP :Actual vs Predicted height
95% CI
Regression (β=0.984)
y = x
Metrics :
R² = -0.411
RMSE = 4.514
Predicted height
Final height
f
Target height :Actual vs Predicted height
95% CI
Regression (β=0.416)
y = x
Metrics :
R² = -0.398
RMSE = 4.494
Predicted height
Final height

Supplementary Fig. 3 Plot of actual vs. predicted height for OLS equations (CV = 5), PH-BP, GP-BP and target height. OLS equation-1: with independent variables of left hand-wrist bone age (BA) based on Greulich-Pyle (GP), radiomic score of femur, father's height and height at menarche; OLS equation-2: with independent variables of BA based on left hand-wrist GP, father's height, height at menarche; OLS equation-3: with independent variables of radiomic score of femur, father's height and height at menarche. PH-BP, BA based on left knee Pyle and Hoerr (PH) atlas and Bayley-Pinneau (BP) method for final height prediction. GP-BP, BA based on left hand-wrist GP and BP method for final height prediction. Target height, defined as a child's mid-parental height, by subtracting 6.5 cm to predict a daughter's height. **a** Plot of actual vs. predicted height for OLS equation-1 (CV = 5); **b** plot of actual vs. predicted height for OLS equation-2 (CV = 5); **c** plot of actual vs. predicted height for OLS equation-3 (CV = 5); **d** plot of actual vs. predicted height for PH-BP method; **e** plot of actual vs. predicted height for GP-BP method; **f** plot of actual vs. predicted height for target height method. CV = 5: 5-fold cross-validation. *OLS* ordinary least squares, *RMSE* root mean square error, *CI* confidence interval
