# Supplementary material for: Final height prediction of girls at menarche: a combined model using left hand and wrist bone age, knee radiomic scores, and clinical characteristics
Source: World J Pediatr. 2025 Dec 13;22(1):129–41. doi: 10.1007/s12519-025-01002-5 (PMC12894113; doi:10.1007/s12519-025-01002-5)
Supplement: Supplementary file 2 — (PDF 241 KB) [file 12519_2025_1002_MOESM2_ESM.pdf]

Supplementary Table 1. Evaluation of predictor robustness: OLS estimation compared with robust regression (Huber's T)

Variables	OLS equation-1	Robust regression-1	OLS equation-2	Robust regression-2	OLS equation-3	Robust regression-3
Intercept						
Estimates (SD)	17.61 (18.08)	28.74 (17.91)	57.76 (6.59)	57.64 (6.53)	-24.06 (21.25)	-17.14 (22.57)
t/*z* (*P*)	0.97 (0.33)	1.61 (0.11)	8.77 (0.00)‡	8.82 (0.00)‡	-1.13 (0.26)	-0.76 (0.45)
Height at menarche						
Estimates (SD)	0.70 (0.04)	0.70 (0.04)	0.74 (0.04)	0.74 (0.04)	0.54 (0.05)	0.54 (0.05)
t/*z* (*P*)	15.80 (0.00)‡	16.15 (0.00)‡	18.42 (0.00)‡	18.46 (0.00)‡	10.93 (0.00)‡	10.39 (0.00)‡
Father's height						
Estimates (SD)	0.09 (0.03)	0.09 (0.03)	0.09 (0.03)	0.09 (0.03)	0.15 (0.04)	0.15 (0.04)
t/*z* (*P*)	3.14 (0.00)‡	16.15 (0.00)‡	2.91 (0.00)‡	3.11 (0.00)‡	4.12 (0.00)‡	3.87 (0.00)‡
BA of GP at menarche						
Estimates (SD)	-1.91 (0.21)	-1.94 (0.21)	-2.01 (0.21)	-2.01 (0.21)	—	—
t/*z* (*P*)	-9.04 (0.00)‡	-9.25 (0.00)‡	-9.56 (0.00)‡	-9.66 (0.00)‡	—	—
Femur radiomic score						
Estimates (SD)	4.07 (1.71)	2.94 (1.70)	—	—	7.09 (2.04)	6.38 (2.17)
t/*z* (*P*)	2.38 (0.02)†	1.73 (0.08)*	—	—	3.48 (0.00)‡	2.95 (0.00)‡

‡, †, and * indicate statistical significance at the 1%, 5%, and 10% levels, respectively, with standard errors reported in parentheses. OLS equation-1 and Robust regression-1: with independent variables of left hand-wrist BA, radiomic score of femur, father's height and height at menarche; OLS equation-2 and Robust regression-2: with independent variables of left hand-wrist BA, father's height, height at menarche; OLS equation-3 and Robust regression-3: with independent variables of radiomic score of femur, father's height and height at menarche. *OLS* ordinary least squares, *BA* bone age, *GP* Greulich-Pyle atlas based on left hand and wrist [7], *SD* standard deviation
