# Supplementary material for: Final height prediction of girls at menarche: a combined model using left hand and wrist bone age, knee radiomic scores, and clinical characteristics
Source: World J Pediatr. 2025 Dec 13;22(1):129–41. doi: 10.1007/s12519-025-01002-5 (PMC12894113; doi:10.1007/s12519-025-01002-5)
Supplement: Supplementary file 3 — (PDF 454 KB) [file 12519_2025_1002_MOESM3_ESM.pdf]

Final height prediction of Chinese girls at menarche: a combined model using left hand and wrist bone age, knee radiomic scores and clinical characteristics

Background

Accurate final height prediction for girls at menarche is important

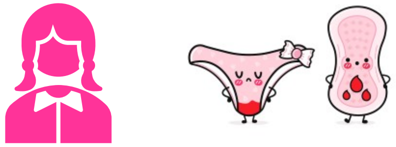

Traditional methods demonstrate limited accuracy

- Greulich-Pyle (GP) and Bayley-Pinneau predictions based on left hand-wrist bone age (BA)
- Target height

Participants and Study Design

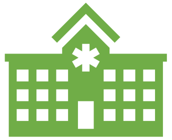

N=173 girls were included as the subjects of our study

Construction of radiomic scores of knee

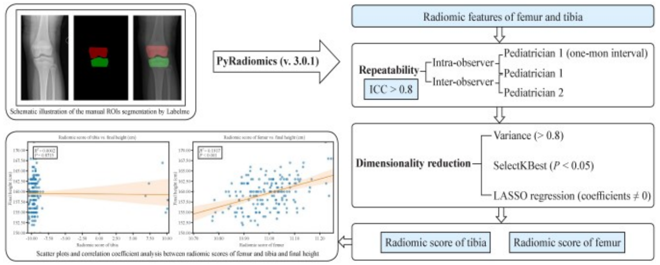

Predictors of final height

- father's height
- mother's height
- height at menarche
- BA of GP
- BA of PH
- radiomic scores of the distal femur and tibia

Stepwise selection

A multilinear equation

- height at menarche
- BA of GP
- radiomic score of the distal femur

($R2 = 0.733$, F statistic $= 115.1$, $P < 0.05$)

Results

The multilinear equation displayed the lowest residuals (residual range: - 5.677 cm to + 6.444 cm) and best Bland-Altman agreement (the mean difference: - 0.01 cm, 95% limits of agreement: - 3.96 to + 3.93 cm).

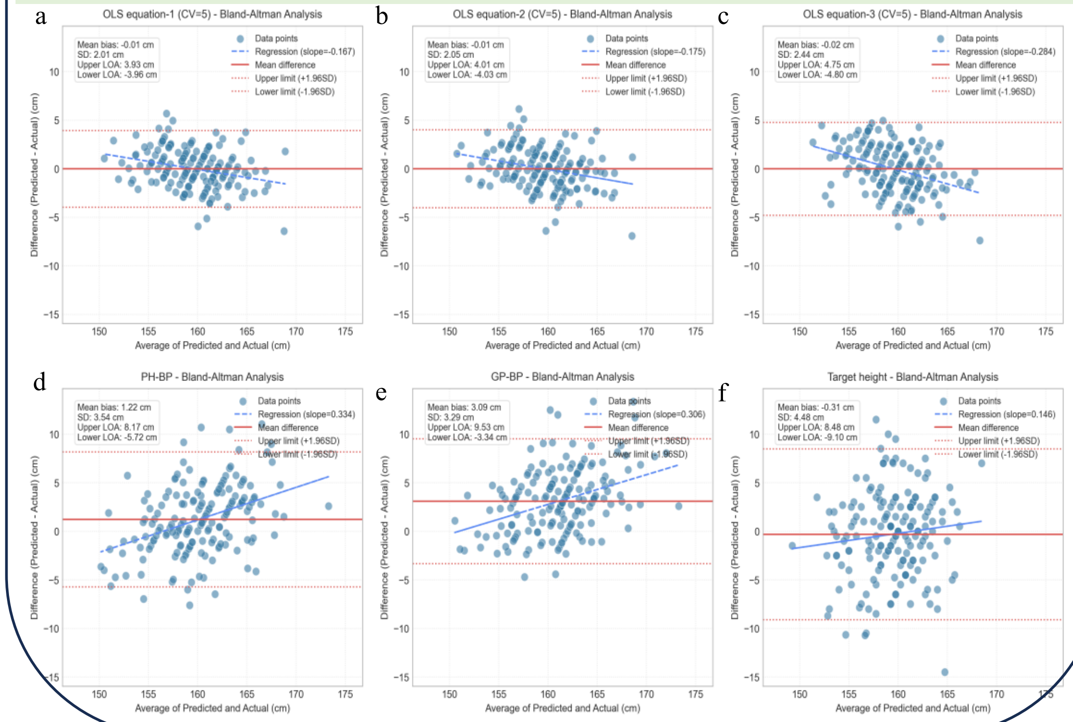

Conclusion:

A robust linear regression model that incorporates knee radiomic scores, BA of GP, height at menarche and father's height demonstrated the best final height prediction in our cohort. This research is an innovative application of radiomic score of the distal femur to final height prediction.
