# Supplementary material for: Final height prediction of girls at menarche: a combined model using left hand and wrist bone age, knee radiomic scores, and clinical characteristics
Source: World J Pediatr. 2025 Dec 13;22(1):129–41. doi: 10.1007/s12519-025-01002-5 (PMC12894113; doi:10.1007/s12519-025-01002-5)
Supplement: Supplementary file 4 — (PDF 253 KB) [file 12519_2025_1002_MOESM4_ESM.pdf]

Supplementary Table Explanations

Table explanations about table 1

Table 1 describes demographic characteristics of 173 study participants.

Table explanations about table 2

Table 2 presents the formulae for radiomic scoring of the distal femur and proximal tibia. The anatomical site refers to the regions being evaluated, specifically the distal femur and proximal tibia.

The PyRadiomics enables extraction of various features, systematically categorized into the following classes:

- First Order Statistics
- Shape-based (2D)
- Gray Level Co-occurrence Matrix
- Gray Level Run Length Matrix
- Gray Level Size Zone Matrix
- Neighbouring Gray Tone Difference Matrix
- Gray Level Dependence Matrix

All feature classes, with the exception of shape can be calculated on either the original image and/or a derived image, obtained by applying one of several filters. In our study, filters specify the image processing techniques used to extract features, which include the original image (Original) as well as high-frequency (Wavelet-H) and low-frequency (Wavelet-L) components derived from wavelet transformation.

A total of 288 radiomic features were extracted from the distal femur and proximal tibia ROIs. Through feature selection, LASSO regression using final height as the dependent variable identified non-zero coefficient features to construct radiomic scores for both regions, thereby constructing radiomic score for both anatomical regions. As

presented in Table 2, the selected features and their corresponding coefficients are listed in detail. *S* represents the linear combination of the intercept and the sum of the products of coefficients and variables. Sign(*S*) ensures that the sign of the radiomic score matches the sign of *S*. Log(|*S*|) is the natural logarithm of the absolute value of *S*. Radiomic score formula = Sign(*S*) × log(|*S*|).

Table explanations about table 3

Table 3 summarizes the ordinary least squares (OLS) regression equations for final height prediction.

As predictors of final height, we used father's height, mother's height, height at menarche, bone age (BA) of Greulich-Pyle (GP), BA of Pyle and Hoerr (PH) and radiomic scores of the distal femur and tibia. Using final height as the dependent variable, we employed a stepwise selection algorithm (with entry $\alpha = 0.05$ and retention $\alpha = 0.10$) to identify significant predictors from seven candidate variables. The analysis yielded four significant predictors for our primary model (OLS equation-1*): height at menarche, BA of GP and radiomic score of the distal femur. To evaluate alternative predictive combinations, we also developed other two OLS equations. OLS equation-2 incorporated BA of GP, height at menarche and father's height. OLS equation-3 included radiomic score of the femur, height at menarche and father's height.

For each equation, we calculated the total variance explained by the model, R2 and adjusted R2; F value and P value from the F-test of overall significance in regression. In addition, we evaluated model performance using the Akaike information criterion (AIC) and Bayesian information criterion (BIC), which balance model fit with complexity to guard against overfitting. Lower values of AIC and BIC indicate a more parsimonious and better-fitting model. To further assess the contribution of each predictor, we computed partial correlation coefficients. Partial correlation coefficients, which quantify the linear relationship between an

independent variable and outcome while controlling for the effects of other variables in the model, provide insights into the direct association of each predictor with the dependent variable, independent of confounding influences.

To facilitate equation comparison, hierarchical hypothesis testing was conducted. For nested models, extra sum-of-squares F test were conducted to evaluate the statistical significance of incremental variance explained by additional variables, whereas likelihood ratio tests (LRT) were performed to compare the increases in R^2 among equations.

Table explanations about table 4 and table 5

To evaluate the predictive accuracy of three OLS models in comparison with traditional methods (PH-BP, GP-BP, and target height), we employed a 5-fold cross-validation (CV = 5) analysis. This technique is essential for assessing how well a machine learning model generalizes to unseen data while mitigating the risk of overfitting. In 5-fold cross-validation, the dataset is divided into five distinct subsets. The model is trained on four of these subsets and tested on the remaining one. This process is repeated multiple times, with each subset serving as the testing set once. By averaging the performance results from each iteration, we obtain an average estimate of the model's predictive accuracy.

Table 4 presents the predicted final heights derived from three OLS models (CV=5), along with the results from the BP-PH, GP-BP and target height methods. Students' *t*-testing of the means revealed that the predicted final height values derived from both the PH-BP and GP-BP methods showed significant differences with the actual final height ($P < 0.05$). In contrast, the three OLS models and the target height did not differ significantly from actual final height.

Table 5 summarizes the performance of three OLS models (CV=5), as well as traditional methods (PH-BP, GP-BP, and target height) for predicting final height. Key metrics in the table include R^2 values, residual analysis (the minimum, median with interquartile range, and maximum residuals), the percentages of absolute residuals falling within specific ranges ($\leq$ 2 cm, between 2 cm and 5 cm, and $\geq$ 5 cm), root mean square error (RMSE) indicating the average error of predictions, and ratio of performance to deviation (RPD) providing a measure of the model's predictive ability.
